# Evolution of continuing medical education in radiology: on-site vs remote

**DOI:** 10.1186/s13244-024-01764-y

**Published:** 2024-08-01

**Authors:** M. Adriaensen, P. Ricci, H. Prosch, M. Rupreht

**Affiliations:** 1 Department of Medical Imaging, Zuyderland Medical Center, Heerlen, The Netherlands; 2 Department of Radiological, Oncological and Pathological Sciences, Sapienza University of Rome, Rome, Italy; 3 Department of Biomedical Imaging and Image‑Guided Therapy, Medical University of Vienna, Vienna, Austria; 4 Radiology Department, UMC Maribor, Maribor, Slovenia; 5 Medical Faculty, University of Maribor, Maribor, Slovenia

**Keywords:** Continuing medical education, Continuing professional development, Live educational events, Electronic learning materials, Radiology

## Abstract

**Objectives:**

To assess the evolution of continuing medical education/continuous professional development (CME/CPD) in *European Radiology* with a particular focus on on-site (live educational events, LEE) vs remote (electronic learning materials, ELM) participation and the impact of the COVID-19 pandemic.

**Methods:**

Results related to CME/CPD of surveys conducted by the Accreditation Council of Imaging (ACI) between 2017 and 2020 are summarized. Additional insights from the survey conducted in spring 2023, exploring online education trends since the start of the COVID-19 pandemic, are presented. Finally, the results of the surveys are correlated with the total number of CME/CPD applications received annually from 2018 to 2022.

**Results:**

Pre-pandemic, 90% of European radiologists supported mandatory CME and unified CME/CPD-system. A trend among younger radiologists towards ELM was observed. Only 20% of employers fully endorsed CME/CPD. In 2020, LEE attendance dropped significantly (95.5–33%), with a simultaneous surge (33–58%) in time spent on ELM. Post-pandemic, the majority (52%) of LEE attendees participated in 1–5 events, whereas the majority (38%) of attendees of live-streamed events participated in 6–20 meetings. Content remains a priority of respondents in all formats: 79% for online, 75% for on-site, and 74% for on-demand. While the assessed quality of LEE remained at the same level (no change (36%) or good/very good (48%)), a considerably higher percentage of respondents noticed the quality of live-streamed events was good/very good (83%).

**Conclusion:**

The majority of European radiologists support mandatory CME and a unified CME/CPD system. Despite the post-pandemic resurgence in LEE, ELM and hybrid events are predicted to gain further prominence.

**Critical relevance statement:**

The CME/CPD system dynamically adapts to evolving professional, technical, and environmental circumstances, with human interaction gaining heightened significance post-COVID-19.

**Key Points:**

- Professionals expressed a desire to return to on-site participation, highlighting its desirability for social interaction.
- Electronic learning materials are poised for continued growth, particularly among younger generations.
- Professionals expressed a desire towards a unified CME/CPD system in Europe.

**Graphical Abstract:**

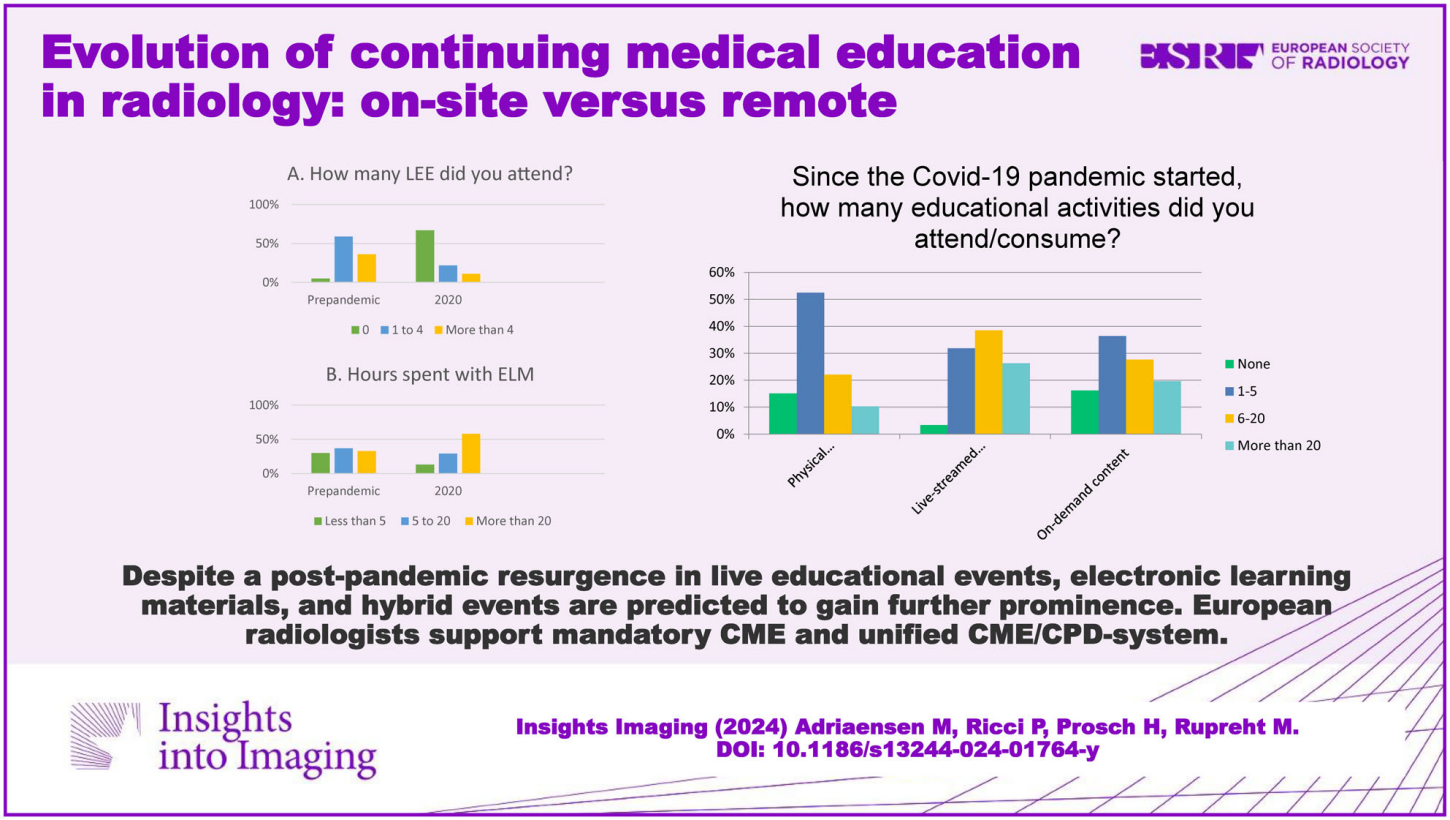

## Introduction

In November 2015, the collaboration between the European Society of Radiology/European Board of Radiology (ESR/EBR) and the European Union of Medical Specialists (UEMS) led to the establishment of the Accreditation Council of Imaging (ACI).

The ACI provides a one-step service for continuing medical education (CME) accreditation, supporting the European Accreditation Council for Continuing Medical Education (EACCME). The ACI conducted several surveys between 2017 and 2020 to gauge the needs of participants and to improve CME for the future [1–5]. In addition, in spring 2023, a survey was performed about trends in CME since the start of the Coronavirus disease 2019 (COVID-19) pandemic.

This article evaluates the performed surveys in the context of the evolution of CME/continuous professional development (CPD) with a special focus on on-site vs remote participation and the influence of the COVID-19 pandemic.

## Methods

This article presents a comprehensive qualitative analysis of the results of the surveys conducted by the ACI from 2017 to 2021, focusing on trends in CME [2–5].

The survey conducted by the ACI in spring 2023 explored changes in education methods since the start of the COVID-19 pandemic. The survey was sent out on the 4th of March 2023 to all ESR full and corresponding members (*n* = 80.623). The survey was open for 2 weeks. Then a reminder was sent, and the members had 2 weeks more to respond. The online web-based software “Surveymonkey” (http://www.surveymonkey.com) was utilized to create and disseminate the survey and collect responses. In accordance with National Health Services Health Research Authority criteria, this study did not require an application for ethical approval [6].

Finally, the results of the surveys are correlated with the total number of applications received annually by the EACCME for live educational events (LEE) and electronic learning Materials (ELM) from 2018 to 2022.

Official approval by the ESR Executive Council for the concept draft of this manuscript was granted to the ACI leadership on the 10th of January 2024.

## Results

### Summary of CME/CPD-related results of prior ACI surveys

#### Survey 2017: accreditation systems in Europe [2]

This survey targeted the presidents of the ESR Institutional member societies (National Radiological Societies and Subspecialty Societies of European countries) and Society delegates to the ESR Education Committee, as well as all individual ESR members from Europe.

All representatives from ESR Institutional Member Societies and 78% of individual ESR members from Europe would support mandatory CME in their countries. Furthermore, 90% of all respondents would be in favour of European CME credits and unified CME/CPD system across European countries.

Regarding the CME format (LEE or ELM), the majority (70% of institutional members and 58% of individual members) preferred a combination of LEE and ELM with emphasis on LEE. Of institutional and individual members, 11% and 21%, respectively, preferred a combination of LEE and ELM with an emphasis on ELM.

#### Survey 2018: the future of CME/CPD [3]

This survey, conducted in 2018, involved sending a questionnaire to all ESR individual members (radiologists and residents) in Europe. It was designed to anticipate upcoming trends regarding CME/CPD activities and processes, additionally using the distribution between generations as a reference.

One thousand one hundred 95 responses were received. In contrast to the 2017 survey [2], the percentage of respondents (45%) indicating a combination of LEE and ELM as the preferred way of earning CMEs was lower in this study. This shift was primarily attributed to a higher percentage (44%) of respondents favouring LEE compared to the previous study (16%). The results also highlighted that the majority (52%) of respondents lacked sufficient information regarding obtaining CME credits. However, positive trends were observed in the perception of the CME/CPD activities as integral to professional improvement, particularly among younger generations. Concerning the future of CME/CPD activities, a significant proportion of respondents (51%) supported the opinion that LEE will continue to play a crucial role in the learning process. Only 20% of respondents indicated that their employers fully supported attendance to LEE by providing financial support and allowing days off. The opinion that ELM will prevail owing to its wide availability and not requiring days off work was expressed by 30% of respondents. Additionally, 12% of respondents believed that accredited webinars would become dominant in the learning process.

**Fig. 1:**
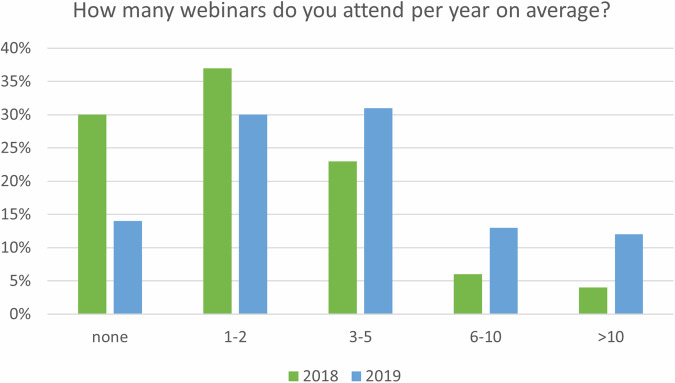
The percentages of participants attending webinars per year from the surveys, conducted in 2018 [3] and 2019 [4]

Analyzing webinar attendance, almost one-third (30%) of all respondents did not attend webinars. Among those who attended, the majority (37%) participated in 1–2 webinars per year, while 23% attended 3–5 webinars, 6% attended 6–10 webinars, and 4% of respondents attended more than 10 webinars per year (Fig. 1). Interestingly, more respondents (44%) did not gain CME credits for attending webinars than those who did (27%).

Although CME-accredited ELM platforms were still not widely accessible, there was an increasing trend among younger radiologists (born between 1981 and 1994, Generation Y group), towards the belief that ELM/webinars would become prevailing CME activities in the future (36% for ELM/17% for webinars). This percentage was considerably higher in comparison to the Boomers/Baby Boomers generation (born between 1946 and 1964) group (25% for ELM/10.42% for webinars), and Generation X group (born between 1964 and 1980) group (32.02% for ELM/9.60% for webinars).

#### Survey 2019: webinars as CME tool [4]

This survey was conducted in October 2019, and questionnaires were distributed to all European members of the ESR (35,000). A total of 732 responses were received.

Among the respondents, 14% indicated that they did not attend webinars at all. Others revealed that they participated in various frequencies, ranging from one to more than 10 webinars per year. The results highlighted a considerably higher attendance of webinars compared to the previous year (Fig. 1).

Notably, 80% of respondents emphasized that CME accreditation would enhance their motivation to attend webinars, further underlining the need for expanded accreditation of webinars to harmonise quality and commercial bias elimination requirements.

#### Survey 2020: impact of COVID-19 on your educational activities [5]

This comprehensive survey was conducted in December 2020 and was sent to all 119,791 ESR Institutional and Associate members. A total of 934 completed the survey, although not all of them answered every question. For each topic, two different parameters were assessed, always referencing both before and since the beginning of the pandemic.

Travel restrictions, lockdowns, and the cancellation of larger gatherings, face-to-face conferences, congresses, and educational courses had been mainly suspended for over a year.

There was a significant decline in LEE attendance. Approximately 60% of the respondents mentioned attending between 1 and 4 LEE per year in the pre-pandemic era. Since the start of the pandemic, 67% of respondents (629) did not attend a single face-to-face meeting.

**Fig. 2:**
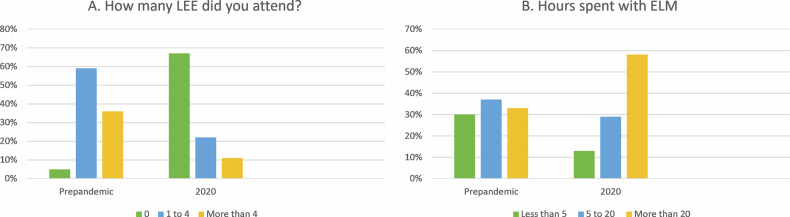
Comparison of attended LEE (**A**) and hours spent with ELM (**B**) pre pandemic and in 2020 [5]

Participants reported an increase in time spent with ELM and webinars. Before the COVID-19 pandemic, exactly one-third of all respondents spent more than 20 h per year with ELM. After spring 2020, this percentage considerably rose to 58% (Fig. 2). The percentage of colleagues attending more than 10 webinars per year increased from 8% in the pre-pandemic era to 41% in the COVID-19 era. Respondents who confirmed having previously spent no time with webinars at all decreased from 32% to 6%.

Users appreciated the fact that on-demand content can be consumed whenever suitable (58%) and the fact that there is no need to take days off or travel (40%).

Regarding financial support for the consumption of ELM, the situation was comparable to face-to-face meetings with 48% paying by themselves, 15% receiving partial financial support, and only 11% receiving full funding from their institution. One-quarter of respondents stated that they only consume ELM if it is free of charge.

The views about the future were mixed. A combination of both LEE and ELM with a preference for LEE (48%) was demonstrated, followed by a mix of both with a preference for ELM. There was a clear preference for LEE in combination with ELM, with 66% of respondents convinced that ELM will gain further ground in medical education and eventually will prevail. Most of the respondents (90%) were convinced that ELM brings savings in costs and time compared to face-to-face meetings.

### Results of survey 2023: online education since the start of the COVID-19 pandemic

This ACI survey, conducted in spring 2023, aimed to assess the evolving landscape of education since the onset of the pandemic, examining the scope and priorities of changes. It delved into the decision-making process for various educational activities, exploring their timing, participant roles, interactivity, and educational benefits in ELM compared to LEE. Five hundred forty-three answers were received, resulting in a response rate of 0.67%.

Question 1 focused on the number of educational activities attended/consumed since the start of the COVID-19 pandemic (Table 1).

**Table 1:** Question 1 of the 2023 survey

Since the Covid-19 pandemic started, how many educational activities did you attend/consume?									
	None		1–5		6–20		More than 20		Total
Physical meetings/conferences/courses	15.10%	82	52.49%	285	22.10%	120	10.31%	56	543
Live-streamed meetings/conferences/courses	3.31%	18	31.86%	173	38.49%	209	26.34%	143	543
On-demand contents	16.21%	88	36.46%	198	27.62%	150	19.71%	107	543
Other (please specify)									9
								Answered	543

Number and type of educational activities attended/consumed since the start of the Covid-19 pandemic

The majority (52%) of those attending LEE participated in 1–5 events, followed by 22%, who attended 6–20 meetings. For live-streamed events, 38% participated in 6–20 meetings, followed by 32%, who attended 1–5 meetings, and 26% attended more than 20 events.

**Fig. 3:**
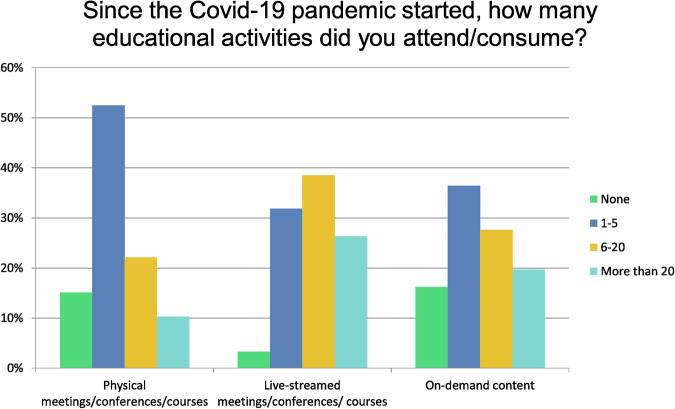
Types and quantity of different educational activities of participants since the start of the COVID-19 pandemic

Regarding on-demand topics, 36% downloaded 1–5 ELM and 28% downloaded 6–20 ELM (Fig. 3).

Questions 2–4 examined priorities when choosing between on-site, live-streamed, and on-demand educational activities during the pandemic, considering factors such as content, speakers, schedule, duration, and CME credits (Table 2).

**Table 2:** Questions 2–4 of the 2023 survey

A. What are the priorities why have you chosen to attend a particular onsite educational activity since the COVID-19 pandemic started?													
	Not a priority		Low priority		Medium priority		High priority		Essential		1		Total
Content	5.16%	28	4.60%	25	15.65%	85	38.49%	209	36.10%	196	0.00%	0	543
Lecturers/speakers/presenters	6.63%	36	6.45%	35	26.34%	143	43.46%	236	17.13%	93	0.00%	0	543
Schedule	7.55%	41	10.68%	58	29.10%	158	39.96%	217	12.71%	69	0.00%	0	543
Duration	11.05%	60	12.52%	68	38.49%	209	30.94%	168	7.00%	38	0.00%	0	543
CME credits	19.34%	105	15.10%	82	26.15%	142	22.47%	122	16.94%	92	0.00%	0	543
Other (please specify)													36
												Answered	543

B. What are the priorities why do you choose to attend a particular live-streamed educational activity since the COVID-19 pandemic started?													
	Not a priority		Low priority		Medium priority		High priority		Essential		None of the above		Total
Content	2.95%	16	2.03%	11	16.02%	87	42.36%	230	36.65%	199	0.00%	0	543
Lecturers/speakers/presenters	4.42%	24	6.26%	34	30.76%	167	41.44%	225	17.13%	93	0.00%	0	543
Schedule	5.16%	28	7.55%	41	29.47%	160	41.07%	223	16.76%	91	0.00%	0	543
Duration	10.13%	55	10.68%	58	35.91%	195	31.49%	171	11.79%	64	0.00%	0	543
CME credits	18.78%	102	12.71%	69	26.70%	145	23.94%	130	17.86%	97	0.00%	0	543
Other (please specify)													12
												Answered	543

C. What are the priorities why do you choose to complete a specific on-demand content since the COVID-19 pandemic started?													
	Not a priority		Low priority		Medium priority		High priority		Essential		None of the above		Total
Content	6.45%	35	4.42%	24	14.92%	81	34.62%	188	39.59%	215	0.00%	0	543
Lecturers/speakers/presenters	8.66%	47	6.45%	35	29.65%	161	37.75%	205	17.50%	95	0.00%	0	543
Schedule	20.99%	114	15.29%	83	23.02%	125	26.52%	144	14.18%	77	0.00%	0	543
Duration	18.42%	100	17.31%	94	30.76%	167	22.84%	124	10.68%	58	0.00%	0	543
CME credits	21.92%	119	15.47%	84	23.02%	125	20.81%	113	18.78%	102	0.00%	0	543
Other (please specify)													12
												Answered	543

Priorities when choosing between on-site (A), live-streamed (B), and on-demand (C) educational activities during the pandemic, considering factors such as content, speakers, schedule, duration, and CME credits

**Fig. 4:**
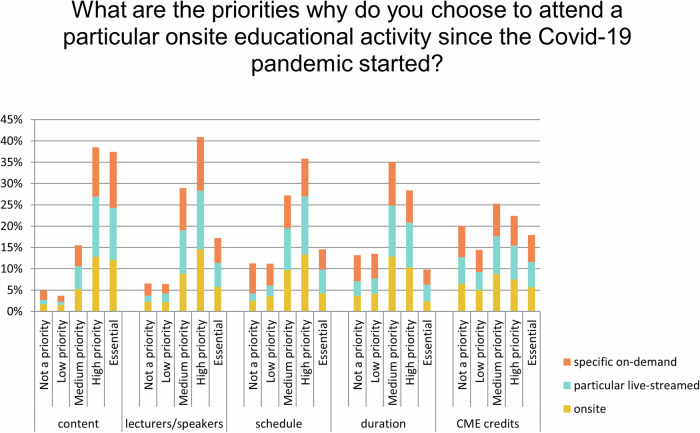
Priorities when choosing on-site, live-streamed and on-demand educational activities

The combined essential or highest priorities of respondents were, in all cases, content: 79% for online, 75% for on-site, and 74% for on-demand content. Speakers were considered essential or highest priority when choosing on-site events (61%), online events (59%), and on-demand content (55%). The schedule was a common priority for live-streamed events (58%), and on-site events (53%). It was, however, less important for on-demand content (41%). CME credits were less prioritized when choosing either on-site event (39%), online events (42%), or on-demand content (34%) (Fig. 4).

Comments regarding reasons to attend on-site events included: networking, hands-on and location. For live-streamed events, common reasons were avoiding travel and travel costs, while easier scheduling was the main reason for prioritizing on-demand content.

Question 5 focused on the quality of educational activities since the beginning of the pandemic (Table 3).

**Table 3:** Question 5 of the survey 2023

Compared to before the COVID-19 pandemic—How do you rate the quality of educational activities since the pandemic started?													
	Poor		Fair		No change		Good		Very good		None of the above		Total
Physical meetings/conferences/courses	3.13%	17	12.71%	69	36.46%	198	30.39%	165	17.31%	94	0.00%	0	543
Live-streamed meetings/conferences/courses	2.03%	11	7.73%	42	7.73%	42	49.91%	271	32.60%	177	0.00%	0	543
On-demand contents	2.21%	12	7.00%	38	16.21%	88	44.20%	240	30.39%	165	0.00%	0	543
Other (please specify)													9
												Answered	543

Quality of educational activities since the beginning of the pandemic

**Fig. 5:**
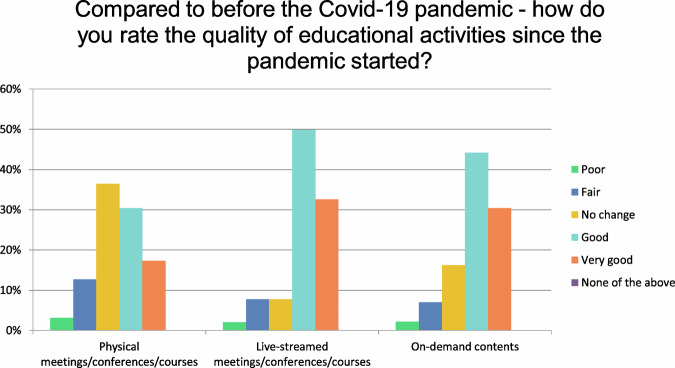
The quality of various educational activities since the pandemic started

Respondents assessed the quality of LEE since the beginning of the pandemic compared to the quality of LEE before the pandemic, with 36% indicating no change and 48% describing it as good/very good. In contrast, a considerably higher percentage of respondents (83%) described the quality of live-streamed events as good/very good compared to the quality before the pandemic. Similarly, the majority (75%) rated the quality of on-demand events as good/very good compared to the quality before the pandemic, indicating an increase in the quality of ELM content since the beginning of the pandemic (Fig. 5).

Question 6 inquired about the timing of consuming live-streamed and on-demand content. The majority of respondents (60%) consumed live-streamed events outside working hours, while an even greater percentage (75%) did the same for on-demand events.

Regarding attendance roles (Question 7), most respondents (60%) were mainly attendees in physical, as well as live-streamed (66%) events.

The interactivity between attendee and speaker/moderator during live-streamed events (Question 8) was rated positively by the majority (59%), with responses indicating good, very good, or excellent experiences. Interestingly, some comments highlighted increased audience participation during live-streamed events, emphasizing the possibility of anonymous questions via Q&A or chat.

**Fig. 6:**
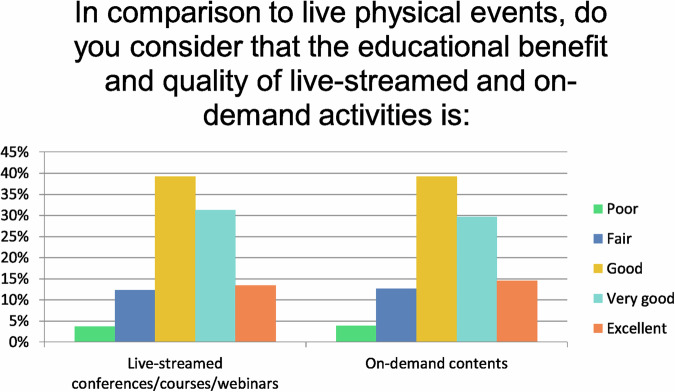
The educational benefits and quality of live-streamed and on-demand activities compared to live physical events since the pandemic started

In comparison to LEE (Question 9), the majority of participants rated the benefits and quality of live-streamed (84%), as well as on-demand events (83%) as good, very good, or excellent, compared to on-site events (Fig. 6).

Several comments highlighted the positive educational aspects of hybrid or online events. For instance: “In my opinion, all conferences should be hybrid and able to be watched on demand for several months after the event. Life is getting more and more expensive. Life events are good for networking etc. Improving knowledge and skills is best done in a quiet environment (i.e. not large noisy conference lecture rooms). And on demand is the best because then the lecture can even be stopped and repeated.” “To study two ways have been proven effective: 1. repetition and 2. reproduction. Both are often easier online (on-demand > live); for the theoretical knowledge, I often prefer online lessons, despite less interaction… There is still a place for physical events.” “The online discussions tend to be much more lively and fruitful than during the physical events because the attendees can type the questions instead of having to stand up and speak in front of an audience, hence the option to type questions (e.g. on smartphones) should be an option for physical events, too.”

### Yearly EACCME application numbers from 2018 to 2022

Contrary to expectations, three years after the outbreak of the COVID-19 pandemic, LEE is thriving, as indicated by the statistics presented by the EACCME on January 14, 2023, in the UEMS Advisory Council (Table 4).

**Table 4:** Yearly EACCME applications from 2018 to 2022

Year	2018	2019	2020	2021	2022
LEE	2031	2318	1490	1973	2156
ELM	144	186	262	281	269

*EACCME* European Accreditation Council for Continuing Medical Education, *LEE* live educational event, *ELM* electronic learning materials

While we have not yet reached the same level of well-being as, in 2019, 2022 has demonstrated a great recovery and a willingness to return to LEE.

Regarding the ELM, the COVID-19 pandemic outbreak has led to a substantial increase in a number of ELM applications.

## Discussion

CME plays a vital role in healthcare and medical education, with most European countries mandating participation [2]. The consistent 25% of respondents across the studies conducted in 2017 [2] and 2019 [4], stating that CME acquisition in their countries remained voluntary, suggests that this situation has persisted over time. Additionally, a substantial majority of European radiologists express support for mandatory CME [2]. A recurring, and even decreasing, low percentage of respondents note that their educational activities receive full support from their institutions [3, 5], underscoring the need for national associations to encourage institutions to fulfil their responsibilities in providing CME/CPD. Results from two surveys, performed in 2020 and 2023, indicate that a similar approach to funding and protected time for ELM, as seen for LEE, is desirable.

The evolving environment, marked by an increasing routine workload and exposure to a growing number of scientific publications, LEE and ELM content, presents a challenge in selecting the right content for participants.

Surveys conducted by the ACI since 2017 consistently reveal a preference for a combination of LEE and ELM, with 66% of respondents convinced that e-learning will continue to gain ground in medical education [5]. This trend is especially noticeable among younger radiologists, compared to older generations [3]. Another positive trend observed in younger generations is the perception of CME/CPD activities as integral to professional improvement [3]. Additionally, unified, simple, intuitive software/platform solutions for online activities (both live streams and on-demand content) would further facilitate online education.

Respondents are also convinced that ELM provides costs and time savings compared to face-to-face meetings. The lower percentage of respondents (45%) favouring the combined method in the 2018 survey [3] compared to the 2017 survey (82% for institutional members and 80% for individual members) [2] might be attributed to a potential decrease in the initial high expectations for ELM, in line with the Gartner hype cycle [7].

In 2019, the ACI already identified webinars as a potential growing educational format. The subsequent outbreak of the COVID-19 pandemic sparked a significant trend toward online education resources, primarily comprising webinars and on-demand resources, as along with virtual LEE. The EACCME and ACI adapted to this new scenario, enabling providers to postpone or convert LEE to ELM. According to the perception of users, the CME system appears to have reacted adequately to the pandemic to meet their demands but does not replace human interaction [8].

Ensuring the physician workforce is adequately prepared to tackle future pandemics, as well as increasing environmental concerns, related to travel, demands a focus on refining the objectives of medical education programs and promoting hybrid events. [9]. In addition, blended learning, combining traditional classroom and online methods to harness the strengths of each, is poised for greater adoption [10]. Nevertheless, as stated in the ACI survey from 2018, we still need to keep in mind that one of the main reasons given by the majority of respondents who declared LEE as a preferred way of earning CME credits is human interaction. Direct formal and informal communication between colleagues and friends in the radiology profession is considered the key determinant, at both an evolutionary and social level, and it is this that makes us what we really are—human beings [3].

The analysis in this paper has several limitations:

Surveys employed different methodologies and targeted similar but not always the same populations: a survey [2] targeted, besides presidents of the ESR Institutional member societies and Society delegates to the ESR Educational Committee, all individual ESR members from Europe. The survey [3] was distributed only to ESR members (radiologists and residents) in Europe. Survey [4] targeted all European members of the ESR (*n* = 35,000), whereas survey [5] was sent to all ESR Institutional and Associate members (*n* = 119,791). The survey conducted in the year 2023 was sent to ESR full and corresponding members (*n* = 80,623). Other than the numbers of recipients, the quoted percentages represent the accessible data of the prior surveys, which, in the majority, lack absolute numbers. Despite seeking information about similar topics, questions in the presented studies were not repeated verbatim. Therefore, conducting a quantitative comparative statistical analysis was not feasible. While the response rate is far from satisfactory (2018: 3.4%, 2019: 2.2%, and 2020: 1.15%), we believe that the received answers provide an overview of the situation among ESR full members. The declining percentage of respondents may be attributed to an increasing number of annual survey invitations per recipient. Only one survey [3] systematically targeted different age groups and different definitions of age groups were used in studies [3] and [5], although they were still similar enough to allow rough comparisons.

## Conclusion

As shown during the pandemic, the CME/CPD system dynamically adapted to evolving professional, technical, and environmental circumstances, with human interaction gaining heightened significance post-COVID-19. Despite a post-pandemic resurgence in LEE, ELM, and hybrid events are predicted to gain further prominence, particularly among younger generations. The majority of European radiologists support mandatory CME and a unified CME/CPD system. Therefore, further efforts towards unifying the CME/CPD systems in Europe should be encouraged.

## Data Availability

The dataset used and analyzed is available from the corresponding author upon reasonable request.

## Abbreviations

ACI: Accreditation Council of Imaging
CME: Continuing medical education
COVID-19: Coronavirus disease 2019
CPD: Continuous professional development
EACCME: European Accreditation Council for Continuing Medical Education
EBR: European Board of Radiology
ELM: Electronic learning materials
ESR: European Society of Radiology
LEE: Live educational event
UEMS: European Union of Medical Specialists
